# Fungal keratitis after small incision lenticule extraction (SMILE): a case report and review of the literature

**DOI:** 10.1186/s12348-021-00256-0

**Published:** 2021-09-03

**Authors:** Mohammad Soleimani, Ali A. Haydar

**Affiliations:** Ocular Trauma and Emergency Department, Farabi Eye Hospital, Tehran University of Medical Sciences, South Kargar Street, Qazvin Square, Tehran, 1336616351 Iran

**Keywords:** Infectious keratitis, Fungal keratitis, *Aspergillus*, SMILE, Penetrating keratoplasty

## Abstract

**Purpose:**

To report a case of perforated fungal keratitis after small incision lenticule extraction (SMILE) treated with penetrating keratoplasty (PKP).

**Methods:**

Case report and literature review.

**Results:**

A 41-year-old woman presented with culture-proven unilateral fungal keratitis 4 days after uneventful SMILE. Her visual acuity was hand motion. The patient was treated with voriconazole irrigation (50 μm/0.1 ml) of the pocket and intrastromal voriconazole injection, in addition to systemic and topical antifungals. Despite aggressive management and decreased infiltration, the cornea was perforated and subsequently treated with PKP.

**Conclusions:**

Infectious keratitis after SMILE is unusual. To our knowledge, this is the first report of perforated fungal keratitis post-SMILE. PKP eradicated the infection.

## Introduction

Infectious keratitis (IK) is a rare yet devastating complication after refractive surgery. A recent meta-analysis reported the risk of IK post keratorefractive surgery as 4 per 10,000 eyes [1]. Small incision lenticule extraction (SMILE) is a relatively new flapless procedure that take benefit from femtosecond laser to cut a precise intrastromal lenticule, which is then extracted via a small keyhole incision [2]. Only few cases of IK post-SMILE have been reported [3–9]. We report an unusual severe unilateral fungal keratitis post-SMILE. The ulcer was refractory to medical treatment and required penetrating keratoplasty (PKP).

## Case report

**Fig. 1:**
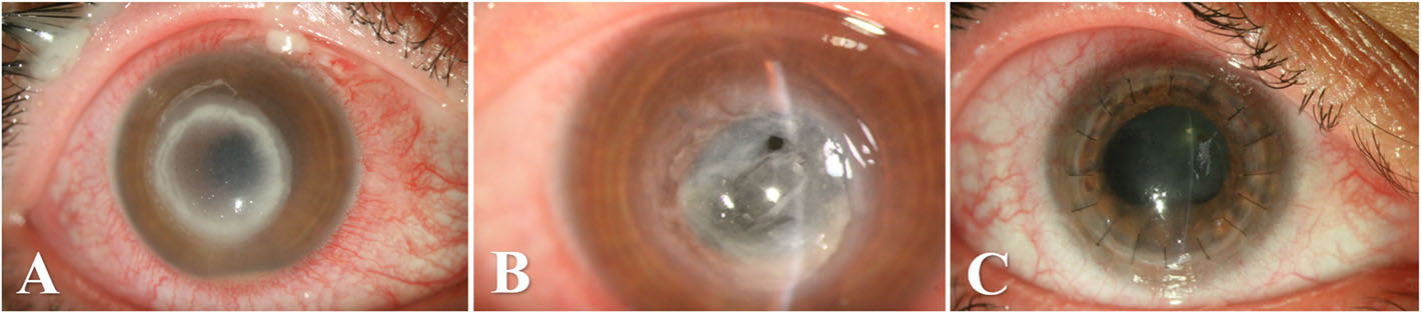
Slit photographs of the right eye with post-SMILE keratitis. **A**, A dense paracentral ring infiltrates taking the shape of the small pocket created during SMILE procedure, on postoperative day 4. Corneal edema and epithelial defect are also seen. **B**, Severe extensive corneal thinning, and perforation. **C**, Tectonic penetrating keratoplasty (PKP) of the right eye

A 41-year-old woman presented to our emergency department with pain and redness in her right eye (RE) for three days. Her past medical history was unremarkable. Four days ago, she underwent uneventful bilateral SMILE procedure for myopia. Her preoperative refractive errors were RE − 3.0 sph and left eye (LE) -3.0 sph − 0.5 cyl 180 axis. The superior cap depth was set at 120 μm, and the depth of the side cut was set at 2 mm. Postoperatively, she was prescribed topical levofloxacin (5 mg/ml) and betamethasone (0.1%) eyedrops every 6 h. Her best-corrected visual acuity (BCVA) in her RE was hand motion (HM) and in her left eye (LE) 20/20. The external examination of the RE showed upper eyelid swelling and protective ptosis. On slit-lamp exam, moderate conjunctival injection and paracentral corneal infiltrate measuring 5 × 5 mm associated with central corneal edema and an overlying epithelial defect were noted (Fig. 1A). A hypopyon which height was 0.2 mm was also seen. The LE exam revealed a clear interface.

**Fig. 2:**
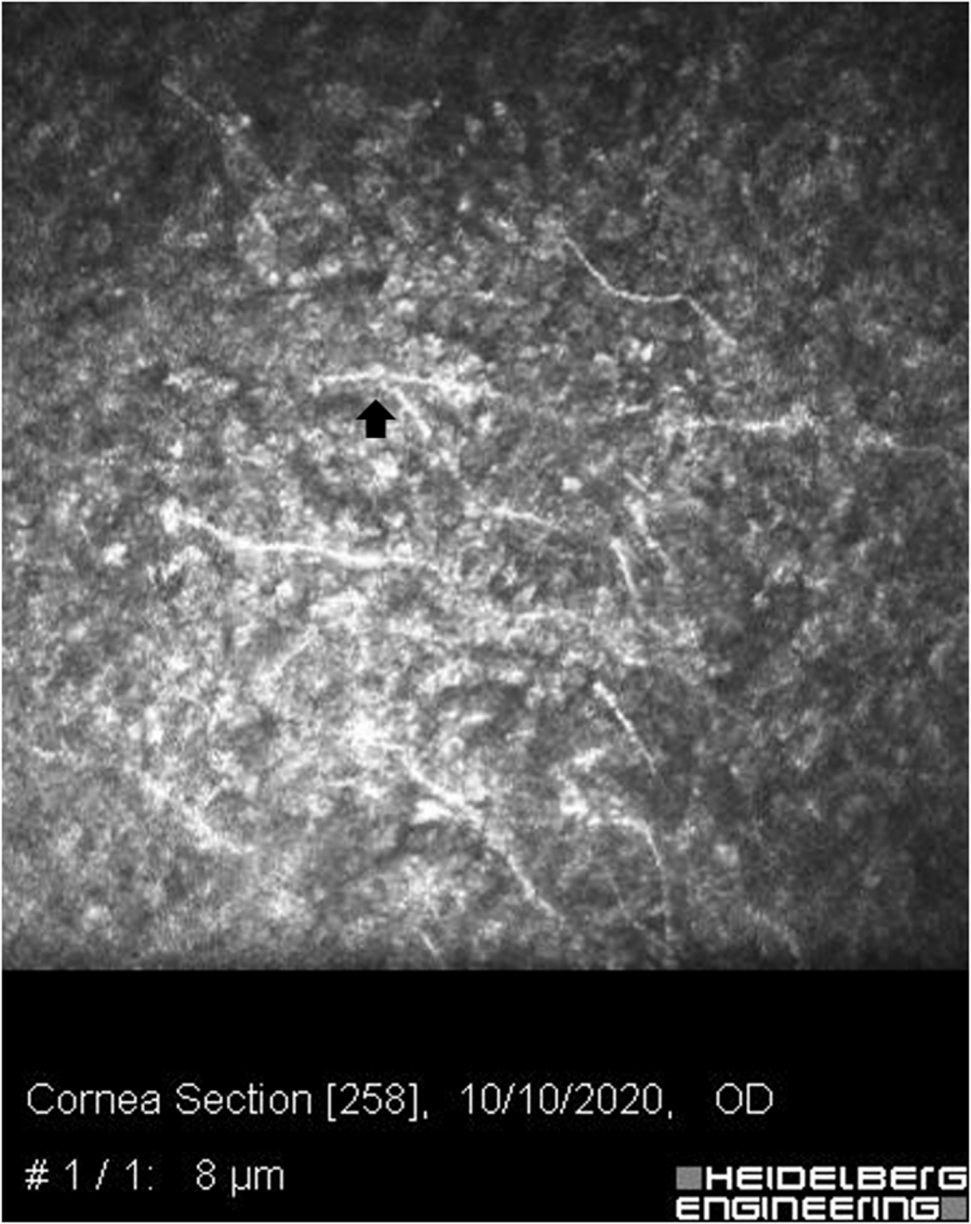
In vivo confocal microscopy (IVCM) of the right eye. Hyphal structures (arrow) are branching and interlocking septate elements

**Fig. 3:**
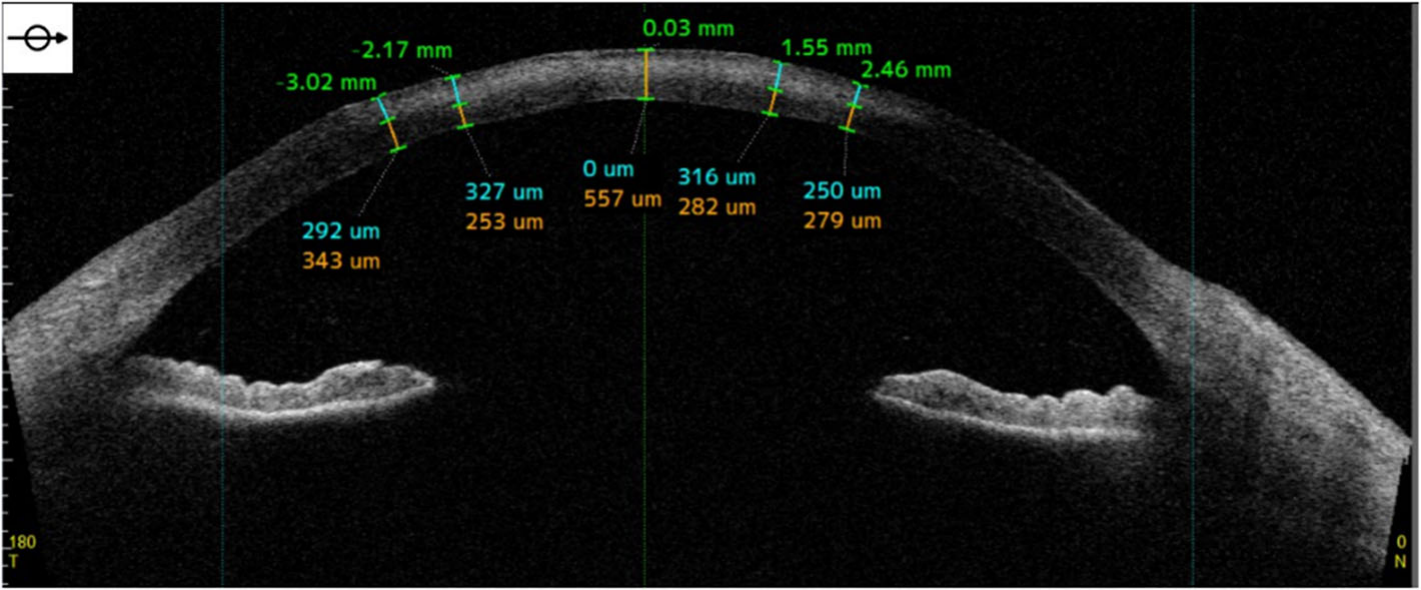
Anterior-segment optical coherence tomography (AS-OCT) of the right eye showing infiltrates and increasing hyperreflectivity in the stromal bed. The numbers above the cornea correspond to eccentricity from the central cornea in mm; numbers below the cornea represent the depth of the infiltrates in the stromal bed in um

Corneal scrapping was performed for microscopic Gram staining that revealed mycelia, and inoculation on Sabouraud and chocolate agars. The patient was admitted and an urgent intrastromal and pocket injection of vancomycin (1 mg/0.1 ml) and voriconazole (50 μg/0.1 ml) was performed—to cover for methicillin resistant *S. aureus* and potential fungi pathogens. After detecting hyphae in the smear, topical voriconazole (10 mg/ml) and levofloxacin (5 mg/ml) were started at a loading dose of every 5 min for the first hour, then every hour. Oral itraconazole (100 mg) every 12 h was also administered. Her antibiogram was sensitive to voriconazole, itraconazole, amphotericin and natamycin. The antifungal susceptibility test using voriconazole and natamycin (Sigma-Aldrich, St. Louis, MO, USA) was done using the E-test method and it was interpreted based on the Clinical and Laboratory Standards Institute (CLSI) M38 3rd ed. [10, 11] *C. parapsilosis* (ATCC 22019) was chosen as a quality control strain in every run. *Aspergillus flavus* strain showed sensitivity to voriconazole with a minimum inhibitory concentration (MIC) of 0.125 μg/mL, and to natamycin (MIC = 0.5 μg/mL). Topical homatropine (2%) was prescribed for cycloplegia. Oral doxycycline (200 mg/daily) and vitamin C (1 g/daily) were added to promote corneal healing. The culture results revealed *Aspergillus* species. A confocal microscope (HRT 3-RCM; Heidelberg Engineering GmbH, Dossenheim, Germany) illustrated the septate branching and interlocking hyphae (Fig. 2). An anterior-segment optical coherence tomography (AS-OCT) (CASIA2; Tomey, Nagoya, Japan) displayed the depth of infiltrations extending deeply to the stromal bed (Fig. 3).

After 10 days, clinical improvement was appreciated as the density of the infiltration was reduced, and the hypopyon resolved. The patient was discharged on fortified voriconazole (10 mg/ml) and levofloxacin (5 mg/ml) eye drops and oral itraconazole (100 mg). She was followed periodically. However, during the healing process, and due to poor compliance, the patient suffered from severe corneal thinning that led to perforation (Fig. 1B). Because of the severe tissue loss and thinning, a PKP procedure was inevitable (Fig. 1C). An 8.5-mm donor graft was used. There was no need to perform lensectomy. The culture of the corneal specimen also showed *Aspergillosis* species. Topical tacrolimus (0.03%) eyedrops every 12 h were added postoperatively to the previous antifungal regimen. Topical steroids every 6 h was started 1 month postop. The patient final BCVA was 20/40 with a refraction of + 3 sph − 7 cyl 135 axis.

## Discussion

SMILE is a newer and less invasive procedure than laser-assisted in-situ keratomileusis (LASIK). IK is a vision-threatening complication after refractive surgery. The incidence and management of IK after LASIK are well documented. We believe that IK post-SMILE may be underreported. Gram-positive bacteria are associated with early-onset post-LASIK IK, whereas fungal and atypical mycobacteria are found in late-onset IK [12]. The safety and efficacy of SMILE is well established and similar to LASIK [2, 13]. The management of IK post-SMILE is more challenging due to the intrastromal closed interface, which is susceptible to rapid spread of infection and is difficult to access in comparison with LASIK flap.

We have reviewed the literature and found a total of ten patients reported to have had post-SMILE IK [3–9]. Table 1 summarizes the case reports of post-SMILE IK. Two large cohort studies investigated the safety of SMILE procedure. Ivarsen et al. reported 5 out of 1800 eyes that developed interface infiltrates [6], and Vestergaard et al. detected only 1 out of 279 eyes [9]. No specific pathogen was isolated in either study. In 2016, Chehaibou et al. reported the first culture-proved case of post-SMILE IK^4^. All but one case was reported in females. All patients presented within 10 days postoperative. The keratitis was bilateral in two cases. All patients were successfully treated with variable visual outcomes (20/50 to 20/20). The described treatments were interface wash and collagen cross-linking with photoactivated riboflavin (PACK-CXL), in addition to fortified eyedrops. Ganesh et al. safely used a combined PACK-CXL and interface wash approach [5].

**Table 1:** Previous case reports of post-SMILE infectious keratitis

Author	Age/Sex	Onset	Pathogen	Characteristic of infiltrates	Management	Outcome (BCVA)
Chehaibou 2016^4^	39/M	Day 2	*S. pneumoniae*	OU: multiple white, at the cap	• Interface wash: povidone-iodine, vancomycin • Fortified antibiotics drops: ticarcillin, gentamicin, and vancomycin	At 3-month OD: CF 50 cm → 20/32 OS: HM → 20/25
Chan 2017^3^	18/F	Day 5	*S. haemolyticus* and *warneri*	OD: paracentral, anterior cap	• PACK-CXL • Fortified antibiotics drops: vancomycin, gentamicin	At 2-week OD: 20/50 → 20/20
Liu 2018^7^	21/F	Day 8	*M. abscessus*	OD: multiple, paracentral, within the cap OS: temporal interface	• Interface wash: moxifloxacin • Fortified antibiotics drops: imipenem, amikacin, moxifloxacin, clarithromycin • Oral clarithromycin	At 4-month OD: 20/32 → 20/32 OS: 20/132 → 20/50
Sachdev 2019^8^	20/F	Day 1	*Aspergillus* species	OD: focal, paracentral, involving the interface	• Interface wash: voriconazole • Fortified antifungals drops: voriconazole and natamycin	At 3-month OD: 20/45
Ganesh 2020^5^	42/F	Day 2	*Staphylococcus* *aureus*	OS: superficial, mid-periphery	• PACK-CXL • Interface wash: vancomycin, moxifloxacin • Fortified antibiotics drops: vancomycin, cefotaxime	At 3-month OS: 20/20

SMILE: small incision lenticule extraction; M: male; F: female; OU: both eyes; OD: right eye; OS: left eye; BCVA: best-corrected visual acuity; CF: counting fingers; HM: hand motion; PACK-CXL: collagen cross-linking with photoactivated riboflavin

To our knowledge, we report the first culture-proven perforated fungal (*Aspergillus*) keratitis post-SMILE in the literature. Our patient underwent uneventful bilateral SMILE and had no risk factor or health problems. She presented on postop day 4 complaining of unilateral pain and redness. The ulcer started with paracentral ring infiltration that progressed to deep central infiltrates involving the stromal bed. Despite aggressive management with fortified eyedrops, interface wash and close follow-up, the keratitis led to severe corneal thinning and perforation. The patient underwent therapeutic PKP to eradicate the infection and preserve the global integrity.

Multiple predisposing factors for IK post-SMILE can be postulated including surgical hygiene, surgeon’s experience, environmental conditions, and periocular infections. The intrastromal pocket created in SMILE might harbor microorganisms inoculated intraoperatively. Also, popular postop use of corticosteroids eyedrops might facilitate secondary infection. Fungal keratitis is more virulent and tissue damaging compared to bacterial keratitis. A meticulous diagnosis, aggressive therapy, and close follow-up are necessary. Corneal scraping is vital for diagnosing fungal keratitis, however early treatment should not be delayed. Fungi appear to penetrate deeper corneal layers [14]. When compared to antibiotics, current antifungals have a lower tissue penetration [15]. Fungal keratitis has greater risk to perforate the cornea than bacterial keratitis [16].

In conclusion, although IK post-SMILE is rare, it can lead to a devastating visual outcome. A rapid diagnosis and aggressive treatment are essential. Fungal keratitis can be refractory to medical treatment, requiring surgical intervention. PKP is a viable option for perforated fungal keratitis.

## Data Availability

The datasets used and/or analyzed during the current study are available from the corresponding author on reasonable request.

## Abbreviations

IK: infectious keratitis
SMILE: small incision lenticule extraction
PKP: penetrating keratoplasty
RE: right eye
LE: left eye
BCVA: best-corrected visual acuity
HM: hand motion
MIC: minimum inhibitory concentration
AS-OCT: anterior-segment optical coherence tomography
LASIK: laser-assisted in-situ keratomileusis
PACK-CXL: collagen cross-linking with photoactivated riboflavin
